# Elements of Successful Food Sovereignty Interventions within Indigenous Communities in the United States and Canada: a Systematic Review

**DOI:** 10.1016/j.cdnut.2023.101973

**Published:** 2023-07-18

**Authors:** Belinda V. Gutierrez, Damita Kaloostian, Nicole Redvers

**Affiliations:** 1 Department of Population Health, School of Medicine & Health Science, University of North Dakota, Grand Forks, ND, United States; 2 School for the Future of Innovation in Society, Arizona State University, Tempe, Arizona, United States; 3 Schulich School of Medicine & Dentistry, University of Western Ontario, London, Ontario, Canada; 4 Department of Indigenous Health, School of Medicine & Health Science, University of North Dakota, Grand Forks, ND, United States

**Keywords:** food sovereignty, food access, interventions, Indigenous Peoples, United States, Canada, systematic review, Indigenous food sovereignty, food insecurity

## Abstract

Despite inherent resiliency and strengths, Indigenous Peoples in the United States and Canada have been impacted by colonialism, which has led to a loss of land, culture, and identity. Loss of land in particular has had substantial impacts on Indigenous food system practices. Indigenous food sovereignty (IFS) has been determined to be a mechanism for Indigenous communities to build their capacity to address food insecurity. A systematic review methodology was therefore engaged to gather and analyze the currently published literature to date to identify common elements of successful IFS interventions within Indigenous communities in the United States and Canada. We carried out a systematic search of the following electronic databases: Academic Search Premier, Agricola, PubMed, CINAHL Complete, Indigenous Studies Portal, the Native Health Database, SocIndex, PsycInfo, and Google Scholar. The Mixed Methods Appraisal Tool was used to apply a methodologic quality score to the included articles. We used a 2-stage process for article selection with 2 independent reviewers screening the titles and abstracts of articles identified. Relevant databases were initially searched up to June 2022 with an updated search occurring in January 2023. Content analysis was carried out on the included articles using qualitative analysis software. Twenty articles met the inclusion criteria of the review. Four main categories of successful elements within IFS interventions were identified, including *1*) transmission of knowledge and skills within the community through workshops, *2*) cultural connectedness through cultivation practices, *3*) preparation and consumption of traditional foods through community programs, and *4*) community-based partnerships and collaborations. An IFS approach has led to the development of several intervention strategies within Indigenous communities, which have been highlighted in this review. The successful elements identified in this review may serve to support future food sovereignty-related programmatic and intervention development within Indigenous communities.

PROSPERO (number: CRD42022342100).

## Introduction

Indigenous Peoples are resilient peoples, with strong ties to culture and land bases around the world. Despite this, over successive generations, colonization has had devastating impacts on Indigenous Peoples in the United States and Canada, resulting in longstanding consequences to individuals, families, and communities [1,2]. Core to these colonial legacies have been negative changes to traditional food systems that serve as critical structures supporting access and availability of foods, food production, consumption, and the sociocultural aspects that are deeply embedded in the ways of living for Indigenous Peoples [2,3]. Disruptions to traditional food systems have also led, and continue to lead, to social and health issues that permeate the Indigenous lived experience [4].

In the United States and Canada, Indigenous Peoples experience higher rates of food insecurity [4,5]. Food insecurity is defined as a situation where people do not have continuous physical or economic access to nutritious, safe foods [6]. Food insecurity has resulted in the overreliance on government food systems that provide food packages consisting of less-nutritious, highly processed, poor-quality, and calorie-dense foods [2]. Diminished traditional food systems have also led to the creation of food deserts in Indigenous communities where it is difficult to obtain affordable or good-quality fresh food [7]. Currently, many Indigenous communities have diminished access to supermarkets and have become reliant on smaller food and convenience stores, which are more expensive and less likely to provide a range of healthful foods [7]. Food insecurity challenges extend beyond the availability of quality foods; it has resulted in a series of compounding health issues such as obesity, diabetes, and heart disease now prevalent within Indigenous communities [2].

Additional factors have also contributed and interacted within the legacies of colonialism, which have additionally contributed to the destruction of traditional food systems. Industrialization has led to the destruction of land and forests, making it difficult and unsafe for Indigenous Peoples to continue traditional cultivation practices [2]. For example, the establishment of dams within Indigenous communities in the United States resulted in the loss of arable land on the Standing Rock, Cheyenne River, Crow Creek, and Fort Berthold reservations in the Dakotas [8]. The installation of a hydroelectric dam called the Churchill River Diversion negatively impacted the wildlife, natural vegetation, and the basic food resources of O-Pipon-Na-Piwin Cree Nation in northern Manitoba [9]. Natural resources such as water have been contaminated by industrialization, impacting fisheries in areas such as the Akwesasne Mohawk community on the US/Canadian border [2]. Climate change has also impacted traditional food systems through droughts in the southwest area of the United States [10,11]. Lack of water resources impedes agricultural production in communities that rely on these foods for sustenance and to support their local economy [2].

Of important note are the sociocultural components integrated within Indigenous traditional food systems that were altered through colonial beliefs and practices [4,5]. These sociocultural components help shape Indigenous Peoples’ traditional food systems but also ensure that cultural transmission occurs for future generations [11]. The transmission of traditional food knowledge, skills, and the preservation of food is all part of the intergenerational transfer of food-system information. The mode of transmission often occurs through Elders in the community who are knowledge keepers for the next generation. Food systems within Indigenous communities in the United States and Canada also served and continue to serve as communal systems with individuals possessing various roles and responsibilities [12]. For example, both women and men had complementary roles within traditional food systems [13].

Historically, among the Omushkego Cree communities, women were instrumental in the skills of hunting and fishing [14]. Within the Haudenosaunee community, children and Elders contributed to cultivation practices while the men coordinated the clearing of vegetation to make way for new fields [15]. These cross-functional responsibilities within traditional food systems provide balance but also ensure equitable responsibility across the communities [15,16]. Additionally, traditional gardening practices and the strategic placement of planting corn, beans, and squash (referred to as the “Three Sisters”) together symbolize sustained life [8]. Food preservation practices are an integral component of Indigenous foodways ensuring long-term food security. Therefore, the conglomerate of sociocultural components of traditional food practices are significant to the next generation of Indigenous Peoples and also promote the health and wellbeing of communities.

The negative impacts to Indigenous food systems have culminated in a series of challenges, including diminished access and availability to quality foods, limitations in resources critical to cultivating culturally supported food choices, and changes to the ways in which Indigenous Peoples interact with each other and food. As a result, many Indigenous Peoples have begun to reclaim their traditional practices through the embodiment of food sovereignty practices with the vision of promoting self-determination and the decolonization of food systems [2,17]. Food sovereignty itself could be defined as “the right of peoples to healthy and culturally appropriate food produced through ecologically sound and sustainable methods, and their right to define their own food and agriculture systems” [5]. Food sovereignty serves as a mechanism for Indigenous communities to build their capacity to address food insecurity but also ensures people have access to healthy foods in a culturally appropriate manner while supporting positive health outcomes [5]. Several Indigenous communities within the United States and Canada have implemented and identified several principles that promote their own food sovereignty [18]. Previous work has also applied Indigenous food sovereignty (IFS) principles specifically as a metric to gage the impact of interventions [5]. To date, a comprehensive IFS intervention framework does not currently exist.

Although intervention strategies have been highlighted within the literature to promote food sovereignty within Indigenous communities in the United States and Canada [5], a formal synthesis of broad intervention strategies has yet to be completed. Therefore, the overarching goal of our systematic review was to gather and analyze the currently published literature to date to identify common elements of successful food sovereignty interventions within Indigenous communities. Our specific objectives of this systematic review were to *1*) outline current food sovereignty intervention strategies within Indigenous communities in the United States and Canada, and *2*) to determine the elements of successful food sovereignty programs within Indigenous communities in the United States and Canada that may inform future efforts.

### Positionality

It is increasingly expected within academic publishing that in the context of Indigenous Peoples, “nothing about us, without us [19].” With this, positionality of the authors within research and associated publications is an important component for any work that pertains to Indigenous Peoples and communities [19,20]. With this, we clarify our positionality to this work here. The first author (BG) is an aspiring public health scholar and enrolled member of the Tohono O’odham Nation in Arizona. The second author (DK) is an African American global development scholar. The senior author (NR) is an Indigenous health scholar, with membership with the Deninu K’ue First Nation located in Denendeh, otherwise known as the Northwest Territories, Canada. All authors position themselves with the intent to bring strength-based dialogue to academic spaces to pave the way for improved health outcomes in Indigenous communities.

## Methods

We engaged systematic review methodology while following the preferred reporting items for systematic reviews and meta-analysis (PRIMSA) for this review [21]. We preregistered a review protocol on PROSPERO before commencement of the review (#CRD42022342100). Covidence software [22] was used for managing the search process and article screening, and Excel 365 software was used for data extraction.

### Search strategy

Our search strategy was inclusive of key terms in the realm of food sovereignty and food security. An example search strategy can be found in Table 1. Relevant databases were initially searched up to June 2022, with an updated search occurring in January 2023. We carried out the systematic search in the following electronic databases: Academic Search Premier, Agricola, PubMed, CINAHL Complete, Indigenous Studies Portal, the Native Health Database, SocIndex, and PsycInfo. A manual search was conducted in Google Scholar. The Google Scholar manual search process consisted of reviewing 2 pages of articles based on the inclusion criteria, with an additional 2 pages reviewed until no additional articles of relevance appeared. The reference sections of key articles were additionally reviewed to expand our search and further identify relevant articles not found during the initial search steps. Our search was limited to English language articles due to the lack of translation support; however, there were no limits on dates of publication to ensure the broadest scope of the available literature. We included peer-reviewed published articles that examined explicit intervention strategies in any age group that promoted or engaged with food sovereignty within Indigenous populations within the United States and Canada. Qualitative, quantitative, and mixed methods study designs were included in the review.

**TABLE 1:** Sample electronic research database search strategy (PubMed)

Dates	All dates to January 2, 2023
**Language(s)**	English
**Keywords**	(“food sovereignty” OR “right to food” OR "food system" OR "food security" OR "traditional food" OR ("Food Security"[Mesh])) AND (intervention OR program OR initiative OR strategies OR ("Program Development"[Mesh])) AND ("american indian" OR "native american" OR "AIAN" OR "AI/AN" OR “NATIVE Americans” OR ("American Indians or Alaska Natives"[Mesh]) OR “North American Indian” OR amerindian OR tribe OR tribal OR “FIRST Nations of Canada” OR “First Nations” OR “Metis” OR “metis” OR "Métis" OR “métis” OR “Inuit” OR "inuit" OR aboriginal OR ("Indians, North American"[Mesh]) OR Indigenous peoples of the Americas OR ("Indigenous Canadians"[Mesh]))

### Article selection and data extraction

We used a 2-stage process for article selection. Two independent reviewers (BG, DK) screened the titles and abstracts of articles identified during the systematic search. A third reviewer was brought in to resolve discrepancies by discussion (NR). The full text articles were then reviewed to determine final inclusion in the review. One reviewer examined 100% of the identified full-text articles (BG), and a second reviewer cross-checked 10% of the full-text articles to ensure consistency (DK). A third reviewer was brought in to resolve discrepancies by discussion (NR). Eligible studies were then retained for data extraction. The first reviewer extracted the following data, including: source (citation, year of publication), methods (aim of study, study design, type of intervention), participants (number of participants, characteristics of participants, urban or rural participant based, region(s) and country/countries recruited from, study eligibility criteria), measures (measurement tools or instruments, method of analysis), results (key conclusions from study authors), and miscellaneous (funding source).

### Quality appraisal and data analysis

Two independent reviewers (BG, DK) used the Mixed Methods Appraisal Tool (MMAT version 2018) to assess eligible studies and determined an overall methodologic quality score [23]. A third reviewer was brought in to resolve discrepancies through discussion (NR). The MMAT assesses the general quality of qualitative, quantitative, and mixed methods studies with 2 initial screening questions and an additional 5 questions for each article type. Content analysis was then carried out on the included articles to identify key categories as outlined by Elo et al. [24] and further clarified for systematic reviews by Mikkonen and Kääriäinen [25]. Content analysis was carried out using NVIVO 12 software (Release 1.7). One reviewer carried out content analysis on 100% of the articles (BG), and a second reviewer cross checked 50% of the articles to assess for consistency (NR). Ongoing discussions were engaged to confirm continuity and shared understanding to further refine the coding process.

## Results

**FIGURE 1:**
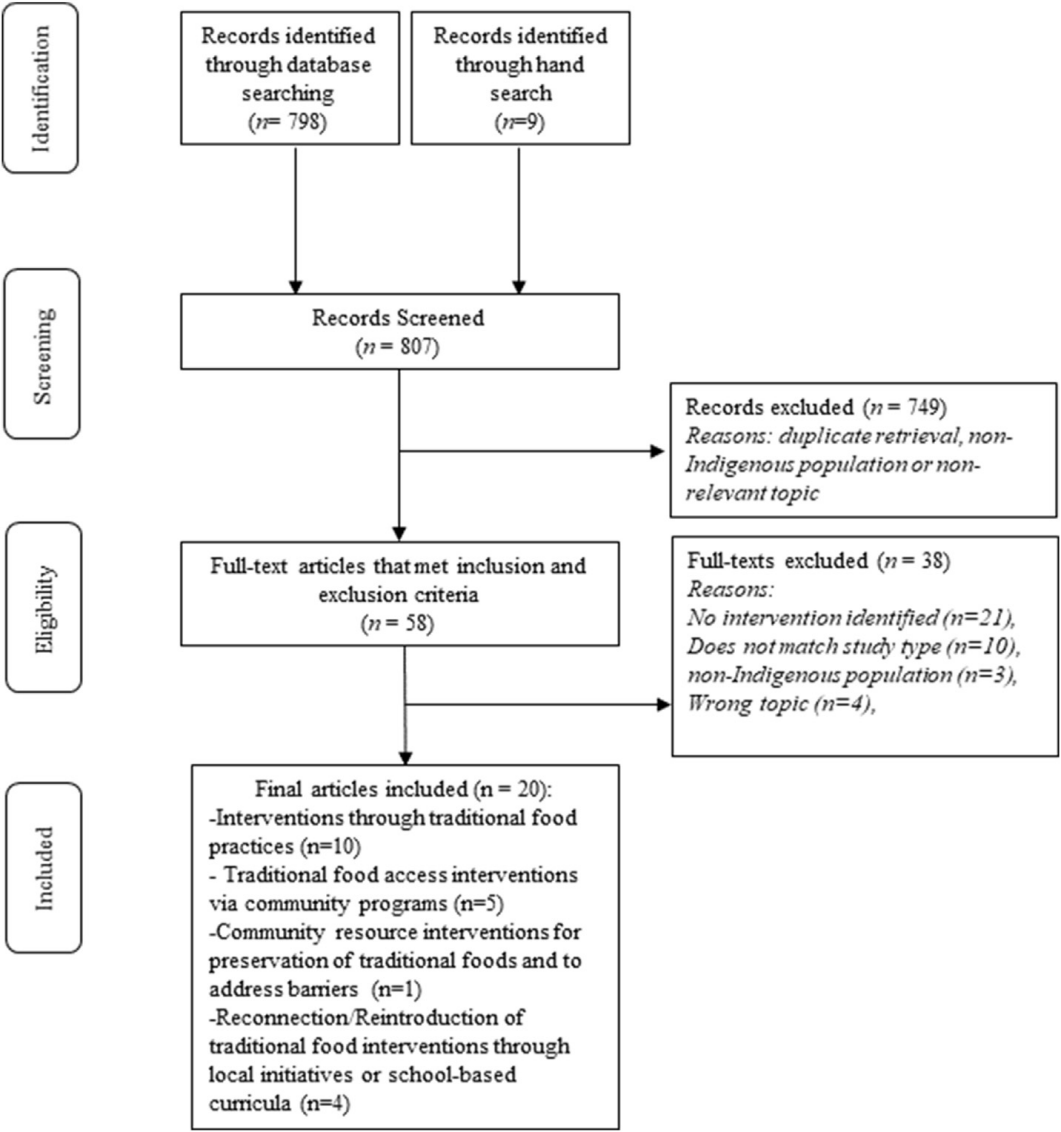
Adapted PRISMA flowchart diagram.

Of the 807 articles identified in the search strategy, 20 articles were ultimately included in the review (see Figure 1). The years of publication of the included articles ranged from 2001 to 2022 with 65% (*n* = 13) of the food sovereignty interventions being based in Canada and 20% (*n* = 4) being based in the United States. Fifteen percent (*n* = 3) of the articles identified intervention strategies in communities that span the US and Canadian borders (see Supplementary Table 1 for the full database of included articles).

The types of interventions carried out in the included articles ranged from garden programs to food preservation interventions (see Table 2). Overall, we found 4 main types of overarching food sovereignty interventions including: interventions through traditional food practices (*n* = 10), traditional food access interventions via community programs (*n* = 5), community resource interventions for preservation of traditional foods and to address barriers (*n* = 1), and reconnection/reintroduction of traditional food interventions through local initiatives or school-based curricula (*n* = 4). The age ranges of participants in the various interventions ranged from 1 year old to 90 years old. Genders were well represented with many participants serving various roles in the community from land managers, harvesters, community members, council members, and community advisory board members. Of the interventions, 55% (*n* = 11) consisted of collaborations among local community organizations and stakeholders within Indigenous communities, while 45% (*n* = 9) of the interventions were implemented as part of collaborations with entities such as public health organizations, governmental organizations, or university partnerships. Results of the MMAT quality appraisal can be found in summary in Table 2 and in detail in Supplementary Table 2.

**TABLE 2:** Characteristics of included studies

	Year	Region	Study design	n	Interventions	Study aims	MMAT quality score∗
Bagelman et al. [27]	2016	Canada	Qualitative	5000	Interventions through traditional food practices	To examine how “Feasting for Change” aimed to empower Indigenous communities in revitalizing traditional knowledge about the healing power of foods.	∗∗∗∗∗
Bagelman et al. [28]	2018	Canada	Qualitative	Youth and Elders from W̱SÁNEĆ First Nations: Tsartlip & Tseycum and other Indigenous territories on Vancouver Island	Interventions through traditional food practices	Indigenous food system revitalization through land and story.	∗∗∗∗∗
Blanchet et al. [36]	2022	Canada/United States	Mixed methods	257	Reconnection/reintroduction of traditional food interventions through local initiatives or school-based curricula	To describe the reach of the Syilx-led reintroduction of Okanagan sockeye salmon intervention and assess its impact on Syilx households’ income-related and cultural food security status.	∗∗∗
Cueva et al. [30]	2020	United States	Qualitative	43	Reconnection/reintroduction of traditional food interventions through local initiatives or school-based curricula	To assess program impact, the “Feast for the Future” to promote access to healthy foods and the transfer of traditional food-based knowledge from farmers/Elders to youth.	∗∗∗∗∗
Cueva et al. [38]	2020	United States	Qualitative	44	Reconnection/reintroduction of traditional food interventions through local initiatives or school-based curricula	To address that gap by describing a community-based obesity-prevention initiative and presenting the perspectives of youth participants in that program, allowing the voices of American Indian youths to describe their perceptions of an obesity-prevention initiative focused on cultural connectedness.	∗∗∗∗∗
Delormier et al. [15]	2018	Canada/United State	Qualitative	22	Interventions through traditional food practices	To share grassroots designed program that integrated the heeds Haudenosaunee teachings, which served as the framework for the planted food-bearing trees and plant program.	∗∗∗
Gendron et al. [26]	2016	Canada	Mixed Methods	119	Interventions through traditional food practices	To explore Indigenous food networks throughout the province of Saskatchewan, Canada, and how they connect people interested in Indigenous foods, and improve food security through Indigenous food activities.	∗∗∗
Gordon et al. [29]	2018	Canada	Mixed Methods	4	Traditional food access interventions via community programs	To examine whether engaging in the Healthy Roots Challenge for 90 d leads to positive outcomes.	∗∗∗∗
Johnson-Jennings et al. [31]	2020	United States	Mixed Methods	27	Interventions through traditional food practices	To reduce obesity among Indigenous children and families who are at risk for homelessness by piloting a gardening health intervention.	∗∗∗∗
Kuhnlein et al. [42]	2001	Canada	Qualitative	500	Traditional food access interventions via community programs	To investigate how an educational intervention program that increases traditional food use can improve health status.	∗∗∗∗∗
McEachern et al. [40]	2022	Canada	Qualitative	89	Traditional food access interventions via community programs	To demonstrate a community engagement model that aimed to enhance access to local, healthy, and traditional foods for youth.	∗∗∗∗∗
Nu et al. [39]	2017	United States	Qualitative	10	Reconnection/reintroduction of traditional food interventions through local initiatives or school-based curricula	To describe formative research and an ongoing collaborative process to design a multilevel nutrition intervention, the Fish-to-School Program that reconnects students to their local food system in a remote Yup’ik community in Western Alaska.	∗∗∗∗∗
Organ et al. [41]	2014	Canada	Qualitative	32	Traditional food access interventions via community programs	To evaluate how one initiative, a community freezer, in Nain, Nunatsiavut supported wild food access for community members.	∗∗∗∗∗
Skinner et al. [33]	2014	Canada	Qualitative	14	Interventions through traditional food practices	To conduct a descriptive case study of the context and process surrounding the implementation of a community greenhouse in a remote, sub-Arctic First Nations community in Ontario, Canada.	∗∗∗∗∗
Stroink et al. [34]	2009	Canada	Mixed methods	20 quantitative respondents. 52 participants from qualitative	Interventions through traditional food practices	To evaluate the process and outcomes of the Learning Garden program using both qualitative and quantitative (survey) methods.	∗∗∗∗
Thompson et al. [35]	2012	Canada	Mixed methods	533	Interventions through traditional food practices	To evaluate food activities in 14 different fly-in or rural communities in Northern Manitoba with the goal of informing future community and policy development.	∗∗∗∗∗
Thompson et al. [32]	2018	Canada	Qualitative	12	Interventions through traditional food practices	To assess the general viability of the hoop house gardening initiative in the community and consider what role it might play in improving local food security.	∗∗∗∗∗
Timer et al. [37]	2019	Canada	Qualitative	25	Interventions through traditional food practices	To evaluate the impacts of a prison garden program to address food insecurity.	∗∗∗∗∗
Wesche et al. [43]	2016	Canada	Qualitative	77	Traditional food access interventions via community programs	To compare and discuss the implications of 2 collaboratively developed, community-based programs to improve capacity for wild food procurement, and identify lessons learned and productive ways forward for communities.	∗∗∗∗∗
Yung et al. [44]	2019	Canada	Qualitative	N/A	Community resource interventions for preservation of traditional foods and to address barriers	To describe how the First Nations Health Authority supported increasing access and knowledge sharing of safely preserved traditional foods through the facilitation of a community champion model.	∗∗∗∗∗

∗ Using the Mixed Methods Appraisal Tool (MMAT) version 2018 for critical appraisal recommends the avoidance of overall global scores. The full MMAT table was therefore added with a record of each quality appraisal parameter in addition to providing the global score here (see Supplementary Data for full table).

Content analysis identified 4 main successful food sovereignty intervention elements among the included article base within Indigenous communities in the United States and Canada, including *1*) transmission of knowledge and skills within the community through hands-on workshops, *2*) enabling cultural connectedness through cultivation practices, *3*) direct consumption and preparation of traditional foods through community programs, and *4*) active community-based partnerships and collaborations.

Transmission of knowledge and skills within the community through hands-on workshops was identified as a common element of food sovereignty interventions within Indigenous communities. Most interventions described the involvement of Elders in knowledge transmission as well as in the direct contribution to programs. For example, one of the intervention workshops described one Elder who shared her Lakota/Dakota knowledge on traditional foods and home remedies found around Standing Buffalo First Nation [26]. Another Elder who hosted a feast workshop also transmitted knowledge sharing through traditional pit cooking and shared methods and techniques with young children and adults in the community [[27], [28]]. Other knowledge holders also contributed to the transmission of knowledge through intervention workshops. In a study by Gordon et al. [29], cooking demonstrations were led by local chefs using traditional foods and traditional activities with community members of all ages.

Enabling cultural connectedness through cultivation practices was identified as an important element of IFS interventions. Several of the interventions clearly identified a sense of cultural connectedness enabled through gardening [[30], [31], [32], [33], [34]]. In a study by Johnson-Jennings et al. [31], during gardening sessions, youth worked alongside Elders who shared ancestral gardening practices and traditional foods through the 7 Anishinaabe teachings. The 7 Anishinaabe grandfather teachings include love, respect, humility, truth, honesty, wisdom, and bravery, which were integrated with notions of healthy land and environment while establishing cultural connections among Indigenous youth in another intervention [[15], [31]]. Another study by Thompson et al. [32] discussed how participants working in hoop gardens described that planting and watching the food grow gave them a feeling of being connected to the food. The integration of ceremony in some interventions also enabled cultural connectedness. A study by Cueva et al. [30] discussed incorporating prayer prior to seed planting where prayer was seen to ensure land nourishment for crop growth. Another intervention included traditional foods and herbs that were planted in gardens where Indigenous teachings and ceremonies were then conducted to connect the participants with the garden and strengthen cultural identity [33]. These gardening interventions provided a sense of being connected with culture but also with the land that produces the foods.

Direct preparation and consumption of traditional foods through community programs was another common element identified within the IFS interventions. Several intervention strategies suggested that traditional food consumption was an important component of programming and therefore included it within their approaches [[30], [42],^44^]. One study by Thompson et al. [35] discussed a community food program in the Nisichawayasihk Cree Nation which distributed “country food” and referenced mammals, fish, plants, berries, waterfowl, and seabirds harvested locally. Direct access to traditional foods enabled communities to become more familiar with their traditional foods but also served as an approach to increase food sovereignty. Another intervention identified through community programming included a study by Blanchet et al. [36], which examined the Syilx-led reintroduction of Okanagan sockeye salmon to the Syilx communities located within Canada. This intervention enhanced the availability of and access to traditional foods while addressing food insecurities. Another intervention identified involved a prison garden program which cultivated traditional foods to donate to rural and remote Indigenous communities in Canada. The implementation of a prison garden intervention in this community resulted in the weekly distribution of the produce to surrounding communities [37]. Direct preparation and consumption of traditional foods through community programs served as a conduit to access traditional foods but also created opportunities for culturally relevant food-based programs within Indigenous communities.

Active community-based partnerships and collaborations were also identified as another common element of successful IFS interventions. Several of the intervention strategies were conducted with internal and external partnerships within or outside Indigenous communities [[30], [36], [37], [38], [39], [40], [41], [42], [43]]. Two of the community garden interventions, for example, were supported by public health organizations [[30], [38]]. The focus of these 2 interventions was aimed at reducing health disparities within Indigenous communities; however, it also served to revitalize traditional food systems [[30], [38], [42]]. University partnerships also served as good collaborators to implement some intervention workshops [[35], [37], [40], [44]]. Through University program support, 2 Indigenous communities established interventions that increased both access to and use of traditional foods at the local level while maintaining cultural continuities [[26], [43]]. Another university partnership between the university and 2 Indigenous communities also provided a mechanism to address the acquisition of critical resources such as fishing supplies and snowmobiles, which are important to the sustainability of traditional food systems in some communities [43].

Community-based collaborations also involved local stakeholders such as schools and governmental agencies who assisted with providing services and resources to community members to support, sustain, or preserve traditional foods. One community-based collaboration project consisted of a local health agency and Indigenous community that implemented community champions [44]. These community champions delivered food-preservation programs; addressed barriers to accessing safe, traditional foods; and increased desire to consume traditional foods. A school in one Indigenous community also implemented their own school-based Fish-to-School Program which reconnected youth to their local food system [39]. Using a community-based participatory approach, the program was developed, implemented, and run by community members, which demonstrated a sense of ownership but also continued the transmission of intergenerational knowledge seen across other food programs.

## Discussion

Our review identified 4 main types of overarching IFS interventions, including those promoting cultural connectedness interventions through traditional food practices; traditional food access interventions via community programs; community resource interventions for preservation of traditional foods and to address barriers; and reconnection/reintroduction of traditional food interventions through local initiatives or school-based curricula. Within these 4 main intervention types, our content analysis identified 4 main elements of successful IFS interventions among the included articles from the United States and Canada. The 4 main elements included transmission of knowledge and skills within the community through hands-on workshops, enabling cultural connectedness through cultivation practices, direct preparation and consumption of traditional foods through community programs, and active community-based partnerships and collaborations. The successful elements identified in this review may serve as a supportive roadmap for developing adaptable local food sovereignty frameworks, and for developing new interventions or initiatives to address IFS in the United States and Canada.

**FIGURE 2:**
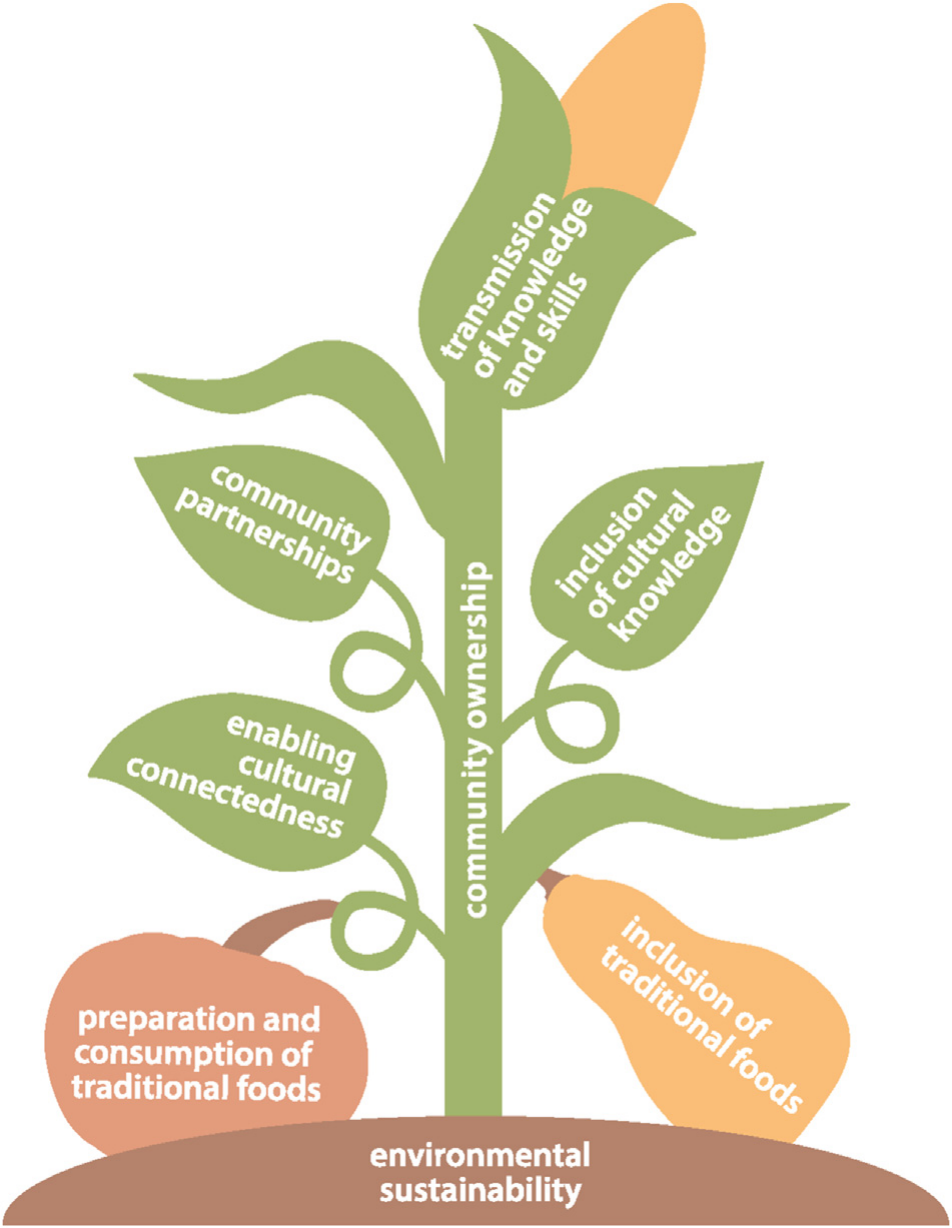
Indigenous food sovereignty (IFS) principles and interconnected intervention elements outlined through the “Three Sisters” (merging current review findings with IFS principles from Maudrie et al. [5]).

Prior work by Maudrie et al. [5] outlined IFS principles that were either explicitly or implicitly referenced in prior work to evaluate a body of intervention literature. Four IFS principles were outlined including: *1*) community ownership, *2*) inclusion of cultural knowledge, *3*) inclusion of traditional foods, and *4*) environmental sustainability of [the] intervention [5]. Three of the IFS principles outlined by Maudrie et al [5] had some alignment with the 4 main elements of successful IFS interventions identified in this review. Considering the synergy between the IFS principles and the intervention elements outlined in this review, we have created an adapted depiction in Figure 2 to accommodate the bridging of principles, processes, and intervention elements. Figure 2 frames the various components of IFS through the lens of the “Three Sisters,” which are corn, climbing beans, and squash planted together in synergy with each other for the benefit of the plants and the stewards of those plants.

The “corn” element of the “Three Sisters” symbolizes the transmission of knowledge and skills within the community. Corn is a sacred plant within many Indigenous communities, being a “must to live” [45]. Corn is embodied within some Indigenous creation stories and ceremonies, and it is a mechanism and a symbol of the transmission of knowledge from generation to generation. Elders have often been the conduit and stewards of corn knowledge, and their role is inherent to many of the interventions identified within this review.

The bean plant within the “Three Sisters” wraps itself around the stalk of the corn plant with an interconnected partnership. The bean plants’ relationship with the corn and the squash (see Figure 2) is based on an understanding of connectedness. Enabling community connectedness and the inclusion of cultural knowledge is only possible through respectful community-based partnerships that are a platform on self-determination and sovereignty (ie, “community ownership” as depicted by the corn stalk). Through an applied lens, without the integration of connectedness and relationships throughout various interventions (the wrapping of the stem of the bean plant), the cultural foundations for such things as gardening, hunting, and farming result in an unstable base for community growth.

Finally, the squash in the “Three Sisters” touches the soil and is the embodiment of culturally grounded interventions. Interventions that are grounded platform the touching, smelling, preparation, and consumption of traditional foods that have sustained communities for millennia.

The preparation and consumption of traditional foods through interventions also serve to provide Indigenous Peoples with access to quality nutritious foods while addressing food insecurity. The “Three Sisters” themselves work together to ensure appropriate nourishment and protection of each other. The plants’ combined presence brings nutrition and growth to themselves in addition to the Indigenous communities that steward these plants.

Overall, there is a lack of published academic literature related to food sovereignty intervention strategies within Indigenous communities in the United States and Canada. This gap limits the sharing of best practices across Indigenous communities that could be helpful for the development of future intervention strategies (especially in low-resource and low-capacity areas) and grant mechanisms. Although assumptions of pan-Indigenous approaches need to be reduced given the diversity of Indigenous Peoples within the United States and Canada, an additional focus on “process” in future work may support the development of a more comprehensive food sovereignty framework for Indigenous Peoples that can be adapted to local contexts. Future questions include: Are there additional process or implementation considerations that can overlay and interact with the successful elements of local food sovereignty interventions already identified? Can Figure 2 be expanded and examined at the local level in varied contexts? Do the successful intervention elements translate to other global Indigenous contexts?

### Limitations

Although the review identified several elements of successful IFS interventions, there were limitations that should be considered. First, some of the articles did not specify participant numbers. Studies that lacked this information made it difficult to determine whether the intervention was effective across large or small population sizes. Secondly, it is also possible that the search strategy that was developed and operationalized did not find all the relevant articles. Given the consistency of categories identified within the published literature base, however, we are confident that the key elements outlined are relevant to IFS interventions within the United States and Canada. Finally, this review examined only published academic articles. It became apparent throughout the review that there may have been some IFS interventions that were mentioned in other article formats, such as opinion pieces referenced from gray literature sources that may be relevant. Given this, future work could expand the review search to be inclusive of community documents and other gray literature to have a more complete understanding of the parameters surrounding the IFS intervention space outside of the published academic literature.

## Conclusion

Forced interruptions to Indigenous food system practices have impeded the ability of many Indigenous Nations to feed themselves. The disruptions to traditional Indigenous food systems have created a sense of loss within many Indigenous communities in the United States and Canada. Food sovereignty has served as an approach to reconnect Indigenous Peoples with their food systems and culture. A food sovereignty approach has led to the development of several intervention strategies among and within Indigenous communities that have been highlighted in this review. The successful food sovereignty elements identified may serve to support future food sovereignty-related programmatic and intervention development within Indigenous communities in the United States and Canada.

## Author contributions

The authors’ responsibilities were as follows – BG, NR: designed research; BG, DK, NR: conducted research; BG, NR: analyzed data; BG, NR: wrote paper; BG, NR: had primary responsibility for final content; and all authors: read and approved the final manuscript.

## Conflict of interest

The authors report no conflicts of interest.

## Funding

The publication of this paper was supported by the Shakopee Mdewakanton Sioux Community through a gift to the University of Minnesota from its Seeds of Native Health campaign.

## Data availability

Data described in the manuscript will be made available upon request pending communication to the authors.

## References

[1] Evans-Campbell T. (2008). Historical trauma in American Indian/native Alaska communities. J. Interpers Violence.

[2] Hoover E. (2017). “You can’t say you’re sovereign if you can’t feed yourself”: defining and enacting food sovereignty in American Indian Community Gardening. Am. Indian Cult. Res. J..

[3] Brouwer I.D., McDermott J., Ruben R. (2020). Food Systems everywhere: improving relevance in practice. Global Food Secur..

[4] Fieldhouse P., Thompson S. (2012). Tackling food security issues in Indigenous communities in Canada: the Manitoba Experience. Nutr. Diet..

[5] Maudrie T.L., Colón-Ramos U., Harper K.M., Jock B.W., Gittelsohn J. (2021). A scoping review of the use of Indigenous food sovereignty principles for intervention and Future Directions. Curr. Dev. Nutr..

[6] Lugo-Morin D.R. (2020). Indigenous communities and their food systems: a contribution to the current debate. J. Ethnic Foods.

[7] Cidro J., Adekunle B., Peters E., Martens T. (2015). Beyond food security: understanding access to cultural food for urban indigenous people in winnipeg as indigenous food sovereignty. Can. J. Urban Res..

[8] Ferreira C., Gaudet J.C., Loukes K.A. (2022). Indigenous women’s worldview in food-related research: rematriating food, bodies and lands. Appl. Physiol. Nutr. Metabol..

[9] Akram-Lodhi A.H. (2013). https://www.tni.org/files/download/15_akramlodi_2013-1.pdf.

[10] Turner N.J., Berkes F., Stephenson J., Dick J. (2013). Blundering intruders: extraneous impacts on two Indigenous food systems. Hum. Ecol..

[11] Drugova T., Curtis K.R., Kim M.K. (2022). The impacts of drought on southwest tribal economies. J. Am. Water Resour. Assoc..

[12] Lemke S., Delormier T. (2017). Indigenous peoples’ food systems, nutrition, and gender: conceptual and methodological considerations. Matern. Child Nutr..

[13] Grey S., Patel R. (2014). Food sovereignty as decolonization: some contributions from Indigenous movements to food system and development politics. Agric. Hum. Val..

[14] Ohmagari K., Berkes F. (1997). Transmission of indigenous knowledge and bush skills among the western james bay Cree women of subarctic Canada. Hum. Ecol..

[15] Delormier T., Marquis K. (2019). Building healthy community relationships through food security and food sovereignty. Curr. Dev. Nutr..

[16] Delormier T., Horn-Miller K., McComber A.M., Marquis K. (2017). Reclaiming food security in the Mohawk community of Kahnawà: ke through Haudenosaunee responsibilities. Matern. Child Nutr..

[17] Coté C. (2016). “Indigenizing” food sovereignty. Revitalizing Indigenous food practices and ecological knowledges in Canada and the United States. Humanities.

[18] Blue Bird Jernigan V., Maudrie T.L., Nikolaus C.J., Benally T., Johnson S., Teague T. (2021). Food sovereignty indicators for indigenous community capacity building and health. Front. Sustain. Food Syst..

[19] Roach P., McMillan F. (2022). Reconciliation and Indigenous self-determination in health research: a call to action. PLOS Global Public Health.

[20] Lock M., McMillan F., Bennett B., Martire J.L., Warne D., Kidd J. (2022). Position statement: research and reconciliation with Indigenous Peoples in rural health journals. Aust. J. Rural Health.

[21] Prisma [Internet]. PRISMA. [cited 2023 Feb 27]. Available from: https://prisma-statement.org/.

[22] Covidence systematic review software, veritas health innovation, Melbourne, Australia. Available at: www.covidence.org.

[23] Hong Q.N., Fàbregues S., Bartlett G., Boardman F., Cargo M., Dagenais P. (2018). The Mixed Methods Appraisal Tool (MMAT) version 2018 for information professionals and researchers. Educ. Inf..

[24] Elo S., Kyngäs H. (2008). The qualitative content analysis process. J. Adv. Nurs..

[25] Mikkonen K., Kääriäinen M., Kyngäs H., Mikkonen K., Kääriäinen M. (2020). The Application of Content Analysis in Nursing Science Research.

[26] Gendron F., Hancherow A., Norton A. (2016).

[27] Bagelman J., Deveraux F., Hartley R. (2016). Feasting for change: reconnecting with food, place & culture. Int. J. Indig. Health.

[28] Bagelman C. (2018). Unsettling food security: the role of young people in indigenous food system revitalisation. Child. Soc..

[29] Gordon K., Lickers Xavier A., Tait Neufeld H. (2018). Healthy roots: building capacity through shared stories rooted in Haudenosaunee knowledge to promote Indigenous foodways and well-being. Can. Food Stud./La Revue canadienne des études sur l’alimentation.

[30] Cueva K., Lovato V., Carroll D., Richards J., Speakman K., Neault N. (2020). A qualitative evaluation of a community based, culturally relevant intervention to promote healthy food access in American Indian Communities. J. Community Health.

[31] Johnson-Jennings M., Paul K., Olson D., LaBeau M., Jennings D. (2020). Ode’imin Giizis: proposing and piloting gardening as an Indigenous childhood health intervention. J. Health Care Poor Underserved.

[32] Thompson H.A., Mason C.W., Robidoux M.A. (2018). Hoop house gardening in the wapekeka first nation as an extension of land-based food practices. Arctic.

[33] Skinner K., Hanning R., Metatawabin J., Tsuji L. (2014). Implementation of a community greenhouse in a remote, sub-Arctic First Nations community in Ontario, Canada: a descriptive case study. Rural Rem. Health.

[34] Stroink M.L., Nelson C.H. (2009). Aboriginal health learning in the forest and cultivated gardens: building a nutritious and sustainable food system. J. Agromed..

[35] Thompson S., Kamal A.G., Alam M.A., Wiebe J. (2012). Community development to feed the family in northern Manitoba communities: evaluating Food Activities based on their food sovereignty, food security, and Sustainable Livelihood Outcomes. Can. J Nonprof. Soc. Econ. Res..

[36] Blanchet R., Willows N., Johnson S., Batal M., Okanagan Nation Salmon Reintroduction Initiatives (2022 Nov 21). Enhancing cultural food security among the syilx okanagan adults with the reintroduction of Okanagan Sockeye Salmon. Appl. Physiol. Nutr. Metab..

[37] Timler K., Varcoe C., Brown H. (2019). Growing beyond nutrition. Int. J. Indig. Health.

[38] Cueva K., Speakman K., Neault N., Richards J., Lovato V., Parker S. (2020). Cultural connectedness as obesity prevention: indigenous youth perspectives on feast for the future. J. Nutr. Educ. Behav..

[39] Nu J., Bersamin A. (2017). Collaborating with Alaska native communities to design a cultural food intervention to address nutrition transition. Prog. Commun. Health Partnersh. Res. Edu. Action.

[40] McEachern L.W., Yessis J., Zupko B., Yovanovich J., Valaitis R., Hanning R.M. (2022). Learning circles: an adaptive strategy to support food sovereignty among First Nations communities in Canada. Appl. Physiol. Nutr. Metabol..

[41] Organ J., Castleden H., Furgal C., Sheldon T., Hart C. (2014). Contemporary programs in support of traditional ways: inuit perspectives on Community Freezers as a mechanism to alleviate pressures of Wild Food Access in Nain, Nunatsiavut. Health & Place.

[42] Kuhnlein H.V., Receveur O., Chan H.M. (2001). Traditional food systems research with Canadian indigenous peoples. Int. J. Circumpolar Health.

[43] Wesche S.D., O’Hare-Gordon M.A., Robidoux M.A., Mason C.W. (2016). Land-based programs in the northwest territories: building indigenous food security and well-being from the ground up. Can. Food Stud..

[44] Yung K., Neathway C. (2020). Community champions for safe, sustainable, traditional food systems. Curr. Dev. Nutr..

[45] United in tradition as peoples of the corn [Internet]. Cultural Survival. [cited 2023Feb27]. Available from: https://www.culturalsurvival.org/publications/cultural-survival-quarterly/united-tradition-peoples-corn.

